# Supplementary material for: Evidence for suppression of immunity as a driver for genomic introgressions and host range expansion in races of Albugo candida, a generalist parasite
Source: eLife. 2015 Feb 27;4:e04550. doi: 10.7554/eLife.04550 (PMC4384639; doi:10.7554/eLife.04550)
Supplement: Supplementary file 1. — List of Arabidopsis thaliana and Brassica spp. accessions assayed in virulence tests with Albugo candida race type isolates AcBoT, Ac2V and AcNc2. DOI: http://dx.doi.org/10.7554/eLife.04550.012 [file elife04550s001.docx]

**Supplementary file 1**

List of *Arabidopsis thaliana* and *Brassica* spp. accessions assayed in virulence tests with *Albugo candida* race type isolates AcBoT, Ac2V and AcNc2.

| **Host** | | | **Pathogen isolate** | | |
| --- | --- | --- | --- | --- | --- |
| Species | Common name | Cultivar / Accession | AcNc2 | Ac2v | AcBoT |
| *B. oleracea* | Purple sprouting broccoli | B&Q311131 | R | nd | S |
| *B. oleracea* | Broccoli | Redhead | R | nd | S |
| *B. oleracea* | Sprouting Broccoli | F1 Corvet | R | nd | nd |
| *B. oleracea* | Sprouting Broccoli | B&Q344981 | R | nd | nd |
| *B. oleracea* | Broccoli | F1 Lord | R | nd | S |
| *B. oleracea* | Calabrese | F1 Belstar | R | nd | S |
| *B. oleracea* | Broccoli | Waltham 29 Organic | R | nd | S |
| *B. oleracea* | Early Purple Sprouting Broccoli | Unwins Early Purple Sprouting | R | nd | S |
| *B. oleracea* | Romanesco | Unwins Romanesco | R | nd | S |
| *B. oleracea* | Early White Sprouting Broccoli | Unwins Early White Sprouting | R | nd | S |
| *B. oleracea* | Autumn Calabrese | Unwins Autumn Calabrese | R | nd | S |
| *B. oleracea* | Broccoli | F1 Olympia | R | nd | S |
| *B. oleracea* | Broccoli | Autumn Spear | R | nd | S |
| *B. oleracea* | Chinese Kale | A12 | R | nd | S |
| *B. oleracea* | Brussels Sprout | Bedford | R | nd | nd |
| *B. oleracea* | Brussels Sprout | F1 Brigitte | R | nd | nd |
| *B. oleracea* | Kale | Maris Kestrel | R | R | S |
| *B. oleracea* |  | 10-12 | nd | R | S |
| *B. juncea* | Oriental Mustard | Cutlass | R | S | R |
| *B. juncea* | Indian mustrad | Czerniac | nd | S | R |
| *B. rapa* | turnip | Just right | R | R | R |
| *A. thaliana* | Mouse-ear cress | Ws-0 | S | R | R |
| *A. thaliana* | Mouse-ear cress | Col-0 | R | R | R |
| *A. thaliana* | Mouse-ear cress | RRS-7 | nd | R | R |
| *A. thaliana* | Mouse-ear cress | Wt-5 | nd | R | R |
| *A. thaliana* | Mouse-ear cress | HR-10 | nd | R | R |
| *A. thaliana* | Mouse-ear cress | Van-0 | nd | R | R |
| *A. thaliana* | Mouse-ear cress | Ksk-1 | R | R | R |
| *A. thaliana* | Mouse-ear cress | Sq-1 | nd | R | R |
| *A. thaliana* | Mouse-ear cress | Pna-17 | nd | R | R |
| *A. thaliana* | Mouse-ear cress | Knox-18 | nd | R | R |
| *A. thaliana* | Mouse-ear cress | Nd-0 | R | R | R |
| *A. thaliana* | Mouse-ear cress | Uod-1 | nd | R | R |
| *A. thaliana* | Mouse-ear cress | Ull2-3 | nd | R | R |
| *A. thaliana* | Mouse-ear cress | NFA-10 | nd | nd | R |
| *A. thaliana* | Mouse-ear cress | Bur-0 | R | R | R |
| *A. thaliana* | Mouse-ear cress | Kin-0 | nd | R | R |
| *A. thaliana* | Mouse-ear cress | Kendl | nd | nd | R |
| *A. thaliana* | Mouse-ear cress | Ler-0 | R | R | R |
| *A. thaliana* | Mouse-ear cress | Ct-1 | R | R | R |
| *A. thaliana* | Mouse-ear cress | Kn-0 | R | R | R |
| *A. thaliana* | Mouse-ear cress | Can-0 | R | R | R |
| *A. thaliana* | Mouse-ear cress | SF-2 | R | R | R |
| *A. thaliana* | Mouse-ear cress | Rsh-1 | nd | nd | R |
| *A. thaliana* | Mouse-ear cress | Mt-0 | R | R | R |
| *A. thaliana* | Mouse-ear cress | No-0 | R | R | R |
| *A. thaliana* | Mouse-ear cress | Rsoh-4 | nd | nd | R |
| *A. thaliana* | Mouse-ear cress | Zu-0 | R | R | R |
| *A. thaliana* | Mouse-ear cress | Wil-2 | R | R | R |
| *A. thaliana* | Mouse-ear cress | Tsu-0 | R | R | R |
| *A. thaliana* | Mouse-ear cress | Wu-0 | R | R | R |
| *A. thaliana* | Mouse-ear cress | Eoli-0 | nd | nd | R |
| *A. thaliana* | Mouse-ear cress | Ns-0 | nd | nd | R |
| *A. thaliana* | Mouse-ear cress | Oy-0 | R | R | R |
| *A. thaliana* | Mouse-ear cress | Rsch-4 | R | R | R |
| *A. thaliana* | Mouse-ear cress | Po-0 | R | R | R |
| *A. thaliana* | Mouse-ear cress | Edi-0 | R | R | R |
| *A. thaliana* | Mouse-ear cress | Hi-0 | R | R | R |
| *A. thaliana* | Mouse-ear cress | Ws-2 | S | R | R |
| *A. thaliana* | Mouse-ear cress | Ws-eds1 | S | S | S |
