# Supplementary material for: Evidence for suppression of immunity as a driver for genomic introgressions and host range expansion in races of Albugo candida, a generalist parasite
Source: eLife. 2015 Feb 27;4:e04550. doi: 10.7554/eLife.04550 (PMC4384639; doi:10.7554/eLife.04550)
Supplement: Supplementary file 2. — List of Arabidopsis thaliana accessions assayed in virulence tests with Albugo candida isolates AcNc2 and Ac2v. DOI: http://dx.doi.org/10.7554/eLife.04550.013 [file elife04550s002.docx]

**Supplementary file 2**

List of *Arabidopsis thaliana* accessions assayed in virulence tests with *Albugo candida* isolates AcNc2 and Ac2v.

| **Abbreviated name** | **Complete name** | **Resistance (R) /Susceptibility (S) to AcNc2** | **Resistance (R) /Susceptibility (S) to Ac2v** |
| --- | --- | --- | --- |
| A-12-402 | Ängsö-12-402 | R | nd |
| A-57-419 | Ängsö-57-419 | R | nd |
| A-59-422 | Ängsö-59-422 | S | nd |
| A-74-430 | Ängsö-74-430 | R | nd |
| A-80-432 | Ängsö-80-432 | R | nd |
| Adal3 | Ådal 3 | S | nd |
| Ade2 | Öde 2 | S | nd |
| Ade3 | Öde 3 | R | nd |
| Ag-0 | Ag-0 | nd | R |
| Ale1-2 | Ale1-2 | R | nd |
| AleA1 | AleA 1 | R | nd |
| Aleda1-1-34 | Aledal-1-34 | R | nd |
| Aleda1-11-63 | Aledal-11-63 | R | nd |
| Aleda1-14-73 | Aledal-14-73 | R | nd |
| Aleda1-17-82 | Aledal-17-82 | R | nd |
| Aleda1-6-49 | Aledal-6-49 | R | nd |
| Algustrum | Algutsrum | R | nd |
| AMA1-17 | ÖMö 1-7 | R | nd |
| An-1 | An-1 | nd | R |
| App1-12 | App1-12 | R | nd |
| App1-14 | App1-14 | R | nd |
| App1-16 | App1-16 | S | nd |
| As-41-1 | Ale-Stenar-41-1 | R | nd |
| As-44-4 | Ale-Stenar-44-4 | R | nd |
| As-50-11 | Ale-Stenar-50-11 | R | nd |
| As-56-14 | Ale-Stenar-56-14 | R | nd |
| As-57-16 | Ale-Stenar-57-16 | R | nd |
| As-59-18 | Ale-Stenar-59-18 | R | nd |
| As-64-24 | Ale-Stenar-64-24 | R | nd |
| As-77-31 | Ale-Stenar-77-31 | R | nd |
| Ba1-2 | Bå1-2 | R | nd |
| Bå3-3 | Bå3-3 | R | nd |
| Bå4-1 | Bå4-1 | R | nd |
| Bå5-1 | Bå5-1 | R | nd |
| Bag1 | Bag 1 | S | nd |
| Ban1 | Bön1 | S | nd |
| Bar1 | Bar 1 | S | nd |
| BAt1 | Böt1 | S | nd |
| BAt4 | Böt4 | S | nd |
| Bay-0 | Bay-0 | nd | R |
| Bil-7 | Bil-7 | nd | R |
| Bil3 | Bil-3 | S | nd |
| Bil5 | Bil-5 | R | R |
| Boo2-3 | Boo2-3 | S | R |
| Bor-1 | Bor-1 | nd | R |
| Bor-4 | Bor-4 | nd | R |
| Br-0 | Br-0 | nd | R |
| Bra-11-135 | Brösarp-11-135 | R | nd |
| Bra-15-138 | Brösarp-15-138 | S | nd |
| Bra-21-140 | Brösarp-21-140 | R | nd |
| Bra-25-142 | Brösarp-25-142 | S | nd |
| Bra-34-145 | Brösarp-34-145 | S | nd |
| Bra-37-149 | Brösarp-37-149 | S | nd |
| Bra-43-152 | Brösarp-43-152 | R | nd |
| Bra-45-153 | Brösarp-45-153 | R | nd |
| Bra-51-157 | Brösarp-51-157 | S | nd |
| Bra-53-159 | Brösarp-53-159 | S | nd |
| Bra-61-162 | Brösarp-61-162 | R | nd |
| Bra-63-163 | Brösarp-63-163 | R | nd |
| Bro1-6 | Brö1-6 | R | nd |
| C24 | C24 | nd | R |
| CIBC-17 | CIBC-17 | nd | R |
| CIBC-5 | CIBC-5 | nd | R |
| CS22491 | CS22491 | nd | R |
| Cvi-0 | Cvi-0 | nd | R |
| Dad1 | Död 1 | R | nd |
| Dad2 | Död 2 | R | nd |
| Dad3 | Död 3 | R | nd |
| Dar10 | Dör-10 | S | nd |
| Dja1 | Dja 1 | S | nd |
| Dja2 | Dja 2 | S | nd |
| Djk3 | Djk 3 | R | nd |
| Dra1-4 | Dra1-4 | R | nd |
| Dra2-1 | Dra2-1 | R | nd |
| Dra3 | Dra3-1 | R | nd |
| Dra3-9 | Dra3-9 | R | nd |
| DraIV 1-14 | DraIV 1-14 | R | nd |
| DraIV 1-5 | DraIV 1-5 | R | nd |
| DraIV 1-7 | DraIV 1-7 | R | nd |
| DraIV 6-16 | DraIV 6-16 | R | nd |
| DraIV 6-35 | DraIV 6-35 | R | nd |
| Eden-1 | Eden-1 | R | R |
| Eden-15 | Eden 15 | R | nd |
| Eden-16 | Eden 16 | R | nd |
| Eden-17 | Eden 17 | S | nd |
| Eden-2 | Eden-2 | S | R |
| Eden-4 | Eden-4 | R | nd |
| Eden-5 | Eden-5 | R | nd |
| Eden-6 | Eden-6 | R | nd |
| Eden-7 | Eden-7 | R | nd |
| Eden-9 | Eden-9 | S | nd |
| Edj 2 | EdJ 2 | S | nd |
| Edj-1 | Eds-1 | R | nd |
| Eds-9 | Eds-9 | R | nd |
| EkN 3 | EkN 3 | R | nd |
| Eks2 | EkS 2 | S | nd |
| Eks3 | EkS 3 | S | nd |
| Est | Est | nd | R |
| Faeb-2 | Fäb-2 | nd | R |
| Faeb-4 | Fäb-4 | nd | R |
| FAL1 | FÄL1 | R | nd |
| FAU4 | FäU 4 | R | nd |
| Fei-0 | Fei-0 | nd | R |
| Fja1-1 | Fjä1-1 | R | nd |
| Fja1-2 | Fjä1-2 | S | nd |
| Fja1-5 | Fjä1-5 | R | nd |
| Fja2-4 | Fjä2-4 | R | nd |
| Fja2-6 | Fjä2-6 | R | nd |
| Fly-2-2 | Fly2-2 | S | nd |
| Fly2-1 | Fly2-1 | R | nd |
| Fly2-2 | Fly2-2 | S | nd |
| FlyA 3 | FlyA 3 | R | nd |
| FrA1 | Frö1 | S | nd |
| FrA3 | Frö 3 | S | nd |
| Fri1 | Fri 1 | S | nd |
| Fri2 | Fri 2 | S | nd |
| Ga-0 | Ga-0 | nd | R |
| GAy-17-198 | Gårdby-17-198 | R | nd |
| GAy-22-213 | Gårdby-22-213 | R | nd |
| Gol-2 | Gol-2 | nd | R |
| Got-7 | Got-7 | nd | R |
| GrA-5 | Grön -5 | R | nd |
| GrAn12 | Grön 12 | S | nd |
| GrAn14 | Grön 14 | S | nd |
| Gro-3 | Gro-3 | S | nd |
| Gu-0 | Gu-0 | nd | R |
| Gul-1-2 | Gul1-2 | R | nd |
| Gy-0 | Gy-0 | nd | R |
| Had 1 | Had 1 | R | nd |
| Had 2 | Had 2 | S | nd |
| Had 3 | Had 3 | R | nd |
| Hag 2 | Hag 2 | R | nd |
| Hal 1 | Hal 1 | R | nd |
| Ham-10-239 | Ham-10-239 | S | nd |
| Ham-13-241 | Ham-13-241 | R | nd |
| Ham-2-228 | Ham-2-228 | S | nd |
| Ham-27-256 | Ham-27-256 | R | nd |
| Ham-6-232 | Ham-6-232 | S | nd |
| Ham-7-233 | Ham-7-233 | R | nd |
| Ham1 | Ham 1 | R | nd |
| Hel3 | Hel 3 | R | nd |
| Hen-16-268 | Hen-16-268 | R | nd |
| Hol A1-1 | HolA1 1 | R | nd |
| Hol A1-2 | HolA1 2 | R | nd |
| HolA2-2 | HolA2 2 | R | nd |
| Hov1-10 | Hov1-10 | R | nd |
| Hov1-7 | Hov1-7 | S | nd |
| Hov3-2 | Hov3-2 | R | nd |
| Hov3-5 | Hov3-5 | R | nd |
| Hovdala-2 | Hovdala-2 | R | nd |
| Hovdala-6 | Hovdala-6 | R | nd |
| HR-5 | HR-5 | nd | R |
| ICE93 | ICE93 | nd | R |
| Kal-1 | Kal 1 | S | nd |
| Kas-2 | Kas-2 | nd | R |
| Kia1 | Kia 1 | R | nd |
| Kni-1 | Kni-1 | S | nd |
| Knox-10 | Knox-10 | nd | R |
| Kondara | Kondara | nd | R |
| Kor1 | Kor 1 | R | nd |
| Kor2 | Kor 2 | R | nd |
| Kor3 | Kor 3 | R | nd |
| Kor4 | Kor 4 | R | nd |
| Kru3 | Kru 3 | R | nd |
| Kulturen1 | Kulturen-1 | S | nd |
| Kva2 | Kva 2 | R | nd |
| Kz-1 | Kz-1 | nd | R |
| Kz-9 | Kz-9 | nd | R |
| Lag1 | Lag 1 | S | nd |
| Lan1 | Lan 1 | R | nd |
| Ler-1 | Ler-1 | nd | R |
| Liarum | Liarum | S | nd |
| Lis-3 | Lis-3 | S | nd |
| LL-0 | LL-0 | nd | R |
| Lov-1 | Löv-1 | R | R |
| Lov-5 | Löv-5 | R | R |
| Lp2-2 | Lp2-2 | nd | R |
| Lp2-6 | Lp2-6 | nd | R |
| Lz-0 | Lz-0 | nd | R |
| Mr-0 | Mr-0 | nd | R |
| Mrk-0 | Mrk-0 | nd | R |
| Ms-0 | Ms-0 | nd | R |
| Mz-0 | Mz-0 | nd | R |
| NÄ¤j2 | Näs2 | R | nd |
| NFA-8 | NFA-8 | nd | R |
| Nok-3 | Nok-3 | nd | R |
| Ny1-13 | Nyl 13 | S | nd |
| Ny1-7 | Nyl-7 | S | nd |
| Omn-1 | Omn-1 | S | nd |
| Omn-5 | Omn-5 | S | nd |
| Omo2-1 | ÖMö2-1 | S | R |
| Omo2-3 | ÖMö2-3 | R | R |
| Or-1 | Ör-1 | R | nd |
| Ost-0 | Ost-0 | R | nd |
| Pna-10 | Pna-10 | nd | R |
| Pro-0 | Pro-0 | nd | R |
| Pu2-23 | Pu2-23 | nd | R |
| Pu2-7 | Pu2-7 | nd | R |
| Puk1 | Puk 1 | S | nd |
| Puk2 | Puk 2 | S | nd |
| Ra-0 | Ra-0 | nd | R |
| RA-17-319 | Röd-17-319 | R | nd |
| Ren-1 | Ren-1 | nd | R |
| Ren-11 | Ren-11 | nd | R |
| Rev-2 | Rev-2 | S | nd |
| Rev-3 | Rev-3 | S | nd |
| Rmx-A180 | Rmx-A180 | nd | R |
| RRS-10 | RRS-10 | nd | R |
| S*Fja1-2 | S*Fja1-2 | S | nd |
| S*Fja1-5 | S*Fja1-5 | R | nd |
| S*Rev-2 | S*Rev-2 | R | nd |
| S*Sparta1 | S*Sparta1 | S | nd |
| S*T1040 | S*T1040 | S | nd |
| S*T1060 | S*T1060 | R | nd |
| S*T1080 | S*T1080 | R | nd |
| S*T1110 | S*T1110 | S | nd |
| S*T1130 | S*T1130 | R | nd |
| S*T510 | S*T510 | R | nd |
| S*T540 | S*T540 | S | nd |
| S*T620 | S*T620 | S | nd |
| S*Tad01 | S*Tad01 | S | nd |
| S*TDR01 | S*TDR01 | R | nd |
| S*TDR03 | S*TDR03 | R | nd |
| S*TDR08 | S*TDR08 | S | nd |
| S*TDR17 | S*TDR17 | S | nd |
| S*TDR18 | S*TDR18 | R | nd |
| S*Tomegap | S*Tomegap | S | nd |
| S*Ull3-4 | S*Ull3-4 | R | nd |
| S294BeL4 | S294BeL4 | R | nd |
| San-2 | San-2 | R | nd |
| Se-0 | Se-0 | nd | R |
| Sha | Sha | nd | R |
| Sim1 | Sim 1 | R | nd |
| Sku-30 | Sku-30 | R | nd |
| Sorbo | Sorbo | nd | R |
| Sparta1 | Sparta-1 | S | nd |
| Spr1-2 | Spr1-2 | R | R |
| Spr1-6 | Spr1-6 | nd | R |
| Spro1 | Spro 1 | R | nd |
| Spro2 | Spro 2 | R | nd |
| Sq-8 | Sq-8 | nd | R |
| Sr:3 | Sr:3 | S | nd |
| St-o | St-0 | R | nd |
| Stabby-13 | Stabby-13 | R | nd |
| Stabby-26 | Stabby-26 | S | nd |
| Ste2 | Ste 2 | R | nd |
| Ste3 | Ste 3 | R | nd |
| Ste4 | Ste 4 | R | nd |
| Stu1-1 | Stu1-1 | R | nd |
| Stu2 | Stu-2 | S | nd |
| T1000 | T1000 | S | nd |
| T1010 | T1010 | S | nd |
| T1020 | T1020 | S | nd |
| T1030 | T1030 | R | nd |
| T1040 | T1040 | S | nd |
| T1050 | T1050 | S | nd |
| T1060 | T1060 | R | nd |
| T1070 | T1070 | R | nd |
| T1080 | T1080 | S | nd |
| T1090 | T1090 | R | nd |
| T1110 | T1110 | S | nd |
| T1120 | T1120 | S | nd |
| T1130 | T1130 | R | nd |
| T1150 | T1150 | S | nd |
| T1160 | T1160 | S | nd |
| T450 | T450 | S | nd |
| T460 | T460 | S | nd |
| T470 | T470 | S | nd |
| T480 | T480 | R | nd |
| T510 | T510 | R | nd |
| T530 | T530 | S | nd |
| T540 | T540 | S | nd |
| T550 | T550 | R | nd |
| T570 | T570 | S | nd |
| T580 | T580 | R | nd |
| T590 | T590 | S | nd |
| T610 | T610 | S | nd |
| T620 | T620 | S | nd |
| T630 | T630 | S | nd |
| T640 | T640 | R | nd |
| T670 | T670 | S | nd |
| T680 | T680 | S | nd |
| T690 | T690 | S | nd |
| T710 | T710 | S | nd |
| T720 | T720 | R | nd |
| T730 | T730 | R | nd |
| T740 | T740 | S | nd |
| T750 | T750 | S | nd |
| T760 | T760 | S | nd |
| T780 | T780 | S | nd |
| T790 | T790 | R | nd |
| T800 | T800 | S | nd |
| T810 | T810 | S | nd |
| T840 | T840 | S | nd |
| T850 | T850 | S | nd |
| T860 | T860 | S | nd |
| T880 | T880 | S | nd |
| T890 | T890 | R | nd |
| T900 | T900 | R | nd |
| T910 | T910 | R | nd |
| T920 | T920 | S | nd |
| T930 | T930 | S | nd |
| T940 | T940 | R | nd |
| T950 | T950 | R | nd |
| T960 | T960 | R | nd |
| T970 | T970 | R | nd |
| T980 | T980 | S | nd |
| T990 | T990 | R | nd |
| TAA03 | TAA 03 | R | nd |
| TAA04 | TAA 04 | R | nd |
| TAA14 | TAA 14 | R | nd |
| TAA17 | TAA 17 | R | nd |
| TAD01 | TÅD 01 | S | nd |
| TAD02 | TÅD 02 | S | nd |
| TAD03 | TÅD 03 | R | nd |
| TAD04 | TÅD 04 | S | nd |
| TAD05 | TÅD 05 | S | nd |
| TAD06 | TÅD 06 | S | nd |
| TAL03 | TÄL 03 | R | nd |
| TAL07 | TÄL 07 | R | nd |
| Tamm-2 | Tamm-2 | nd | R |
| Tamm-27 | Tamm-27 | nd | R |
| TBA01 | TBÖ 01 | S | nd |
| TDr11 | TDr-11 | S | nd |
| TDr13 | TDr-13 | S | nd |
| TDr14 | TDr-14 | S | nd |
| TDr15 | TDr-15 | S | nd |
| TDr16 | TDr-16 | S | nd |
| TDr18 | TDr-18 | R | nd |
| TDr2 | TDr-2 | R | nd |
| TDr22 | TDr-22 | R | nd |
| TDr4 | TDr-4 | R | nd |
| TDr5 | TDr-5 | R | nd |
| TDr7 | TDr-7 | R | nd |
| TDr8 | TDr-8 | S | nd |
| TDr9 | TDr-9 | R | nd |
| TEDEN02 | TEDEN 02 | R | nd |
| TEDEN03 | TEDEN 03 | R | nd |
| TFA02 | TFÄ 02 | R | nd |
| TFA04 | TFÄ 04 | R | nd |
| TFA05 | TFÄ 05 | R | nd |
| TFA06 | TFÄ 06 | R | nd |
| TFA07 | TFÄ 07 | R | nd |
| TFA08 | TFÄ 08 | R | nd |
| TGR01 | TGR 01 | S | nd |
| TGR02 | TGR 02 | S | nd |
| THA03 | THÖ 03 | S | nd |
| THA08 | THÖ 08 | R | nd |
| Ting-1 | Ting-1 | R | nd |
| TNY04 | TNY 04 | S | nd |
| TOM01 | TOM 01 | R | nd |
| TOM02 | TOM 02 | R | nd |
| TOM03 | TOM 03 | R | nd |
| TOM04 | TOM 04 | R | nd |
| TOM06 | TOM 06 | R | nd |
| TOM07 | TOM 07 | R | nd |
| Tomegap-2 | Tomegap-2 | S | nd |
| Tos-31-374 | Tos-31-374 | R | nd |
| Tos-75-382 | Tos-75-384 | S | nd |
| Tos-82-387 | Tos-82-387 | R | nd |
| Tos-93-391 | Tos-93-391 | S | nd |
| Tos-95-393 | Tos-95-393 | R | nd |
| Tottarp-2 | Tottarp-2 | R | nd |
| TRA01 | TRÄ 01 | S | nd |
| Ts-1 | Ts-1 | nd | R |
| Ts-5 | Ts-5 | nd | R |
| Tur3 | Tur 3 | S | nd |
| Tur4 | Tur 4 | S | nd |
| TV-10 | TV-10 | S | nd |
| TV-22 | TV-22 | R | nd |
| TV-30 | TV-30 | R | nd |
| TV-38 | TV-38 | R | nd |
| TV-4 | TV-4 | R | nd |
| TV-7 | TV-7 | R | nd |
| UduI 1-34 | UduI 1-34 | R | nd |
| Ull2-13 | Ull2-13 | S | nd |
| Ull2-5 | Ull2-5 | R | R |
| Ull3-4 | Ull3-4 | R | nd |
| UllA-1 | UllA 1 | R | nd |
| UllA-2 | UllA 2 | R | nd |
| UOD-7 | UOD-7 | nd | R |
| Var2-1 | Vår2-1 | R | R |
| Var2-6 | Vår2-6 | R | R |
| VArA1 | VårA 1 | R | nd |
| Västervik | Västervik | S | nd |
| Vimmerby | Vimmerby | S | nd |
| Vinslöv | Vinslöv | R | nd |
| Wa-1 | Wa-1 | nd | R |
| Wei-0 | Wei-0 | nd | R |
| Yo-0 | Yo-0 | nd | R |
| Yst 1 | Yst 1 | S | nd |
| Yst 2 | Yst 2 | R | nd |
| Zdr I 2-24 | Zdr I 2-24 | R | nd |
| Zdr-1 | Zdr-1 | nd | R |
| Zdr-6 | Zdr-6 | nd | R |
| ZdrI 2-25 | ZdrI 2-25 | R | nd |
