# Supplementary material for: Evidence for suppression of immunity as a driver for genomic introgressions and host range expansion in races of Albugo candida, a generalist parasite
Source: eLife. 2015 Feb 27;4:e04550. doi: 10.7554/eLife.04550 (PMC4384639; doi:10.7554/eLife.04550)
Supplement: Supplementary file 3. — Polymorphisms between Albugo candida race genomic regions verified with Sanger sequencing. DOI: http://dx.doi.org/10.7554/eLife.04550.014 [file elife04550s003.docx]

**Supplementary file 3**

Polymorphisms between *Albugo candida* race genomic regions verified with Sanger sequencing.

| **Contig ID** | **Race** | **Forward primer sequence** | **Reverse primer sequence** | **Product length** | **SNP confirmed?** |
| --- | --- | --- | --- | --- | --- |
| 317 | AcNc2 | Tgtatgttctttatactacagaaggg | atggtttttactttcgtgatca | 220 | YES |
| 850 | AcNc2 | gatacgtacggagtatcggcaa | cagaatgttttcttacagttggc | 230 | YES |
| 524 | AcNc2 | atcttacctggcgaagagtac | ttcaaagttatcccgacgat | 229 | YES |
| 736 | AcNc2 | Atgaggtatattaagatcacttgca | aatctatcaatcccagtaccacat | 190 | YES |
| 52 | AcNc2 | Cactaccaaactcaaaaatgataag | attctttaatttggacggtcc | 323 | YES |
| 202 | Ac2V | tggtcctctttgtaataacgc | tgtcagatacgttacgctca | 202 | YES |
| 37016 | Ac2V | tagagatttattgctctggaag | aaagtcgcccattatttctg | 201 | YES |
| 22779 | Ac2V | cacgaatgcctggtaatctcg | cagcgaaaaaagagccctct | 238 | YES |
| 15350 | Ac2V | accaaaaggctctgtattctgc | gcaaaagtttctgcatttcga | 266 | YES |
| 16524 | Ac2V | gtctacttcagatggttggtgtc | aataggacgtaagcattttgatc | 147 | YES |
| 12496 | AcBoT | tgcaatacataagggatggaaa | gaaagcaagatcgtccgaag | 292 | YES |
| 10351 | AcBoT | Ttgatcagctaacatattgaacacc | gaatccaaagcggctatcag | 246 | YES |
| 28900 | AcBoT | cgcatgagaaagcaaagagc | gacgaaacgagcaccacac | 249 | YES |
| 7809 | AcBoT | ttcatttgtcctctgcaaaca | tgctcgacataattcactgtctt | 290 | YES |
| 7809 | AcBoT | catcatgaccgggaaagaac | gcagggtatgctcttggaga | 243 | YES |
| 348 | AcNc2 | aggaatgccggtctttttct | ttattgcattgccactctcg | 254 | YES |
| 1001 | AcNc2 | ggaaccgtggaaattgaaga | tccgctgatctcattcagag | 158 | NO |
| 75 | AcNc2 | cgggaactgttgattcaggt | ctggaactcatttccgaggt | 245 | YES |
| 194 | AcNc2 | tgattctctcacattgattctgc | gctgcagctgatctaacacag | 201 | YES |
| 252 | AcNc2 | ggaagctttcgtttcagcaa | tgcttgaactgctttgcact | 220 | YES |
| 305 | AcNc2 | cgaattgttccgttcctctc | tcccatgcttcttgttgattc | 246 | YES |
| 349 | AcNc2 | caagaacggccgaattaaaa | tgtagtgcggttgaccagag | 247 | YES |
| 1219 | AcNc2 | gagccagcgccttatttatg | cccaaaacacccaaaagaaa | 226 | YES |
| 46 | AcNc2 | ttccaaccgtgctatcattg | ttcgaatacggtgatttcca | 248 | YES |
| 41 | AcNc2 | ggctccgtaccctgctctat | gctggtttcgttgaggttgt | 192 | YES |
