# Supplementary material for: Evidence for suppression of immunity as a driver for genomic introgressions and host range expansion in races of Albugo candida, a generalist parasite
Source: eLife. 2015 Feb 27;4:e04550. doi: 10.7554/eLife.04550 (PMC4384639; doi:10.7554/eLife.04550)
Supplement: Supplementary file 5. — Details of race-specific primers. DOI: http://dx.doi.org/10.7554/eLife.04550.016 [file elife04550s005.docx]

**Supplementary file 5**

Details of race-specific primers. Primers were designed using Primer3 software based on alignment data generated for all three *A. candida* race genomes and were verified to be race-specific on pure spore DNA of all three races before being approved for use in distinguishing between races in co-infection assays.

| **Ac Race** | **Contig** | **Product size** | **Primer direction** | **Start** | **Stop** | **Primer Seq** | **Tm (°C)*** |
| --- | --- | --- | --- | --- | --- | --- | --- |
| AcNc2 | AcNc2_CONTIG_53_length_108163 | 781 | Fw | 3113 | 3132 | GCCACAGGCAAACTACCAAT | 64.2 |
|  |  |  | Rv | 3893 | 3874 | TTTGCAGGAAGCCTTTCAGT | 64.2 |
| Ac2v | Ac2V_NODE_811_length_18469 | 599 | Fw | 17688 | 17707 | CGACAAACAAGCGAATGAGA | 64.4 |
|  |  |  | Rv | 18286 | 18267 | CCAACTGATGGAACACAACG | 64.5 |
| AcBoT | AcBoT_NODE_18478_length_54490 | 559 | Fw | 51421 | 51402 | AACTTCAGGCTTCACCGAGA | 64.3 |
|  |  |  | Rv | 50863 | 50882 | TTCTGCTTCTCCGCGTTTAT | 64.3 |

* Tm was calculated using the ThermoScientific Tm calculator which uses Modified Breslauer's thermodynamics, dH and dS parameters.
