# Supplementary material for: Evidence for suppression of immunity as a driver for genomic introgressions and host range expansion in races of Albugo candida, a generalist parasite
Source: eLife. 2015 Feb 27;4:e04550. doi: 10.7554/eLife.04550 (PMC4384639; doi:10.7554/eLife.04550)
Supplement: Supplementary file 6. — Details of co-infections assays. DOI: http://dx.doi.org/10.7554/eLife.04550.017 [file elife04550s006.docx]

Supplementary file 6

Details of co-infections. Secondary inoculum was administered 7 days after primary inoculum. Co-infection experiments with AcNc2 + Ac2v on Ws-2 were repeated twice while AcBot + Ac2v on *B. oleracea* as well as Ac2v + Nc2 on *B. juncea* were repeated three times.

|  | **Plant details** | | **Primary inoculum** | | **Secondary inoculum*** | | **Result** |
| --- | --- | --- | --- | --- | --- | --- | --- |
|  | Plant | No. | Treatment | No. of plants | Treatment | No. of plants |  |
| AcNc2 + Ac2V co-infection | Ws*-*2 | 16 | AcNc2 | 8 | Ac2V | 4 | AcNc2, Ac2V present |
|  |  |  |  |  | Water | 4 | AcNc2 only |
|  |  |  | Water | 8 | Ac2V | 4 | No pathogen detected |
|  |  |  |  |  | Water | 4 | No pathogen detected |
|  | Ws-*eds1* | 16 | AcNc2 | 8 | Ac2V | 4 | AcNc2, Ac2V present |
|  |  |  |  |  | Water | 4 | AcNc2 only |
|  |  |  | Water | 8 | Ac2V | 4 | Ac2V only |
|  |  |  |  |  | Water | 4 | No pathogen detected |
| AcBoT + Ac2V co-infection | *B. oleracea* | 12 | AcBoT | 6 | Ac2V | 3 | AcBoT, Ac2V present |
|  |  |  |  |  | Water | 3 | AcBoT only |
|  |  |  | Water | 6 | Ac2V | 3 | Small signal for Ac2V |
|  |  |  |  |  | Water | 3 | No pathogen detected |
|  | Ws-*eds1* | 16 | AcBoT | 8 | Ac2V | 4 | AcBoT, Ac2V present |
|  |  |  |  |  | Water | 4 | AcBoT only |
|  |  |  | Water | 8 | Ac2V | 4 | Ac2V only |
|  |  |  |  |  | Water | 4 | No pathogen detected |
| Ac2V + AcNc2 co-infection | *B. Juncea* | 12 | Ac2V | 6 | AcNc2 | 3 | Ac2V, AcNc2 present |
|  |  |  |  |  | Water | 3 | Ac2V only |
|  |  |  | Water | 6 | AcNc2 | 3 | No pathogen detected |
|  |  |  |  |  | Water | 3 | No pathogen detected |
|  | Ws-*eds1* | 16 | Ac2V | 8 | AcNc2 | 4 | Ac2V, AcNc2 present |
|  |  |  |  |  | Water | 4 | Ac2V only |
|  |  |  | Water | 8 | AcNc2 | 4 | AcNc2 only |
|  |  |  |  |  | Water | 4 | No pathogen detected |
